# Serological analyses against endemic human coronaviruses and SARS-CoV-2 in children and adults using samples collected before the COVID-19 pandemic

**DOI:** 10.1016/j.ijregi.2024.100485

**Published:** 2024-11-06

**Authors:** Yusuke Sayama, Chuan Lo, Hiroki Tomizawa, Mayuko Saito, Michiko Okamoto, Suguru Ohmiya, Hidekazu Nishimura, Hitoshi Oshitani

**Affiliations:** 1 Department of Virology, Tohoku University Graduate School of Medicine, Sendai, Japan; 2 Virus Research Center, Clinical Research Division, Sendai Medical Center, Sendai, Japan

**Keywords:** Seroprevalence, Human coronavirus, SARS-CoV-2, Children, Adults, Japan

## Abstract

**Objectives:**

Four endemic human coronaviruses (HCoVs), HCoV-229E, HCoV-NL63, HCoV-HKU1, and HCoV-OC43, infect humans during childhood and cause the common cold. COVID-19 caused by SARS-CoV-2 leads to mild symptoms in children, possibly owing to the protection conferred by immunity developed during a previous HCoV infection. This study analyzed the seroreactivity of four endemic HCoVs and SARS-CoV-2 in children and adults.

**Methods:**

A total of 747 serum samples (from individuals aged 6 months to 69 years) were collected from 2015 to 2019 before the COVID-19 pandemic in Japan. The samples were tested for immunoglobulin G antibodies against the four endemic HCoVs and SARS-CoV-2 wild-type spike ectodomain proteins using enzyme-linked immunosorbent assay.

**Results:**

The seroprevalence of endemic HCoVs (except HCoV-229E) showed 90% positivity by 3-4 years old, whereas HCoV-229E seroprevalence was observed at 8 years old. Approximately 35% of the samples showed reactivity to SARS-CoV-2 and did not change with age. However, the children's group presented higher antibody levels than the adult group. The sample reactivity against SARS-CoV-2 did not confirm neutralization capability.

**Conclusions:**

The reactive samples against SARS-CoV-2 showed varying antibody levels among different age groups. These findings may contribute to a deeper understanding of the clinical symptoms of COVID-19 and coronavirus diseases.

## Highlights

• Individuals aged <8 years showed >90% seropositivity against endemic coronaviruses.
• Approximately 35% of samples showed immunoglobulin G seropositivity against SARS-CoV-2.
• Antibody levels against SARS-CoV-2 in children were higher than those in adults.
• Reactive samples against SARS-CoV-2 did not confirm a neutralization capability.

## Introduction

Coronaviruses are membrane-enveloped, non-segmented, positive-stranded RNA viruses infecting humans and various animals [1]. The coronavirus genome has approximately 30,000 nucleotides and consists of two large open reading frames (ORFs), ORF1a and ORF1b, which encode nonstructural proteins involved in viral replication and transcription. The remaining several ORFs produce structural and accessory proteins [1,2]. In particular, the spike protein on the virion surface mediates viral entry into host cells and is crucial for treatment with targeted monoclonal antibodies or vaccine development [3].

Seven coronaviruses infect humans: four endemic human coronaviruses (HCoVs) (HCoV-229E, HCoV-NL63, HCoV-HKU1, and HCoV-OC43), severe acute respiratory syndrome coronavirus (SARS-CoV), Middle East respiratory syndrome coronavirus, and SARS-CoV-2 [[4], [5], [6], [7], [8], [9], [10]]. SARS-CoV-2 is the etiological agent of COVID-19.

The four endemic HCoVs usually cause mild to moderate symptoms of the upper respiratory tract infection (e.g. the common cold), and the first infection typically occurs within 5 years of birth in temperate and tropical regions [[11], [12], [13], [14], [15]]. Most children infected with SARS-CoV-2 also experience mild or asymptomatic infections [16]. These clinical features may be associated with the protection conferred by immunity developed during recent endemic HCoV infections. Recently, children have shown strong innate immune responses to SARS-CoV-2 in the nasal mucosa and high spike-specific T-cell responses [17,18]. In contrast, antibodies against SARS-CoV-2 can cross-react with endemic HCoVs in humoral immunity, although they might not have neutralizing capability [11,[19], [20], [21]]. However, these reports about humoral immunity against SARS-CoV-2 have been limited to analyses using samples collected from children and adults after the COVID-19 pandemic. Thus, in this study, we conducted serological analyses of HCoVs (including the four endemic HCoVs and SARS-CoV-2 wild-type [WT]) using samples from children and adults collected before the COVID-19 pandemic to identify immunity levels against HCoVs between children and adults.

## Methods

### Ethics statement

This study was approved by the Tohoku University Graduate School of Medicine Ethics Committee (approval nos. 2022-1-432 and 2022-1-1091).

### Study population

A total of 747 serum samples were collected from two hospitals in Sendai, Miyagi Prefecture, Japan; 517 serum samples were collected from outpatients (aged 6 months to 19 years; children) at a private pediatric clinic between January 2015 and December 2019, and 230 serum samples were collected from hospital staff at regular health checkups (aged 20 to 69 years [adults]) at the Sendai Medical Center in July 2019. The serum samples remaining after the original purpose of investigations were stored at −30°C at the Sendai Medical Center in Japan until further use. SARS-CoV-2–infected serum samples were purchased from a commercial laboratory (RayBiotech, GA, USA) and used as positive controls for serological assays against SARS-CoV-2. All serum samples were inactivated at 56°C for 30 minutes before experiments.

### Immunoglobulin (Ig) G enzyme-linked immunosorbent assay

Enzyme-linked immunosorbent assay (ELISA) was performed as previously described [11,22]. Briefly, recombinant spike ectodomain proteins from HCoV-229E, HCoV-NL63, HCoV-HKU1, HCoV-OC43, and SARS-CoV-2 (WT) were purchased from a commercial laboratory (Sino Biological, Beijing, China). Each protein (1 ng/µl in phosphate-buffered saline [PBS]) was immobilized in a 96-well Immulon 1B plate (Thermo Fisher Scientific, Waltham, MA, USA). After blocking with the BlockAce/blocking reagent (Yukijirushi, Sapporo, Japan), the plates were incubated with serum samples diluted with blocking reagent (1:100), followed by incubation with peroxidase-conjugated anti-human immunoglobulin G (1:5000; Southern Biotech, AL, USA). Optical density (OD) was measured at 450 nm using an EPOCH2 spectrophotometer (BioTek/Agilent Technologies, CA, USA). The background signal from the blank wells (non-antigen–coated) was subtracted from the signals of all the tested samples. A sample was considered positive if the net OD value was above the cut-off value, calculated for each antigen as the mean net OD + 5 × SD of three negative sera and was at least 0.1 [11]. The antibody level was defined as the ratio of the cut-off value (ratio of the OD value of the test sample to the cut-off value calculated from the mean of the negative control values in each plate).

### Plaque reduction neutralization test using SARS-CoV-2 wild-type

The plaque reduction neutralization test was performed as previously described [11,23]. The VeroE6/transmembrane serine protease 2 (TMPRSS2) cell line was purchased from the Japanese Collection of Research Bioresources (Osaka, Japan). VeroE6/TMPRSS2 cells were seeded and incubated in 12-well plates until a cell monolayer was formed. Serum samples were diluted using a plain medium (Dulbecco's Modified Eagle Medium, Thermo Fisher Scientific, 1:10); mixed with an equal volume of medium containing 50-100 plaque-forming units of SARS-CoV-2 (SMC-VC-1) isolated from a patient infected with SARS-CoV-2 on October 29, 2020 and incubated at 37°C for 60 minutes. Subsequently, 100 µl serum/virus mixture was added to each well and the plate was incubated at 37°C for 60 minutes, followed by the addition of 2 ml overlay medium (Avicel and methylcellulose) [23]. After 4 days, the cells were fixed with 4% paraformaldehyde and stained with crystal violet. The number of plaques was manually determined by two investigators, including one who was blinded to the sample layout. The cut-off value for neutralization inhibition was set at 50%.

### Statistical analyses

All statistical analyses were conducted using GraphPad Prism (San Diego, CA, USA) version 10.1.0. Mann–Whitney U tests were used to compare the differences in nonparametric observations between the two groups. Statistical significance was defined as *P* <0.05.

## Results

### Population characteristics

The demographic characteristics of the study are summarized in Table 1. Of the 747 participants included in the study, 162 (21.7%) were aged 0.5-5 years, 308 (41.2%) were aged 6-12 years, 47 (6.3%) were aged 13-19 years, and 230 (30.8%) were adults (aged 20-69 years); 379 (50.7%) were male and 368 (49.3%) were female.

**Table 1:** Demographic characteristics and anti-HCoVs spike IgG detection of this study.

Age (years)	Male	%	Female	%	Total
0.5−2	18	60	12	40	30
3	17	56.7	13	43.3	30
4	26	53.1	23	46.9	49
5	22	41.5	31	58.5	53
6	32	58.2	23	41.8	55
7	28	48.3	30	51.7	58
8	23	46	27	54	50
9	17	45.9	20	54.1	37
10	29	63	17	37	46
11	19	52.8	17	47.2	36
12	11	42.3	15	57.7	26
13−19	20	42.6	27	57.4	47
20−29	25	50	25	50	50
30−39	25	50	25	50	50
40−49	25	50	25	50	50
50−59	25	50	25	50	50
60−69	17	56.7	13	43.3	30
Total	379	50.7	368	49.3	747

IgG positive	Antigen	Value	%		
		
	HCoV-229E	649	86.9		
	HCoV-NL63	720	96.4		
	HCoV-HKU1	722	96.7		
	HCoV-OC43	737	98.7		
	SARS-CoV-2 (WT)	258	34.5		
All negative		1	0.13		

HCoV, human coronaviruses.

### Seroprevalence of HCoV-229E, HCoV-NL63, HCoV-HKU1, and HCoV-OC43

We performed ELISA using recombinant spike ectodomain proteins to detect IgG antibodies against the four endemic HCoVs. Of 747 serum samples tested, only one (0.13%) sample from an 8-month-old child tested negative for IgG antibodies against HCoVs. Notably, 649 (86.9%) serum samples tested positive for HCoV-229E, 720 (96.4%) for HCoV-NL63, 722 (96.7%) for HCoV-HKU1, and 737 (98.7%) for HCoV-OC43 (Table 1).

**Figure 1:**
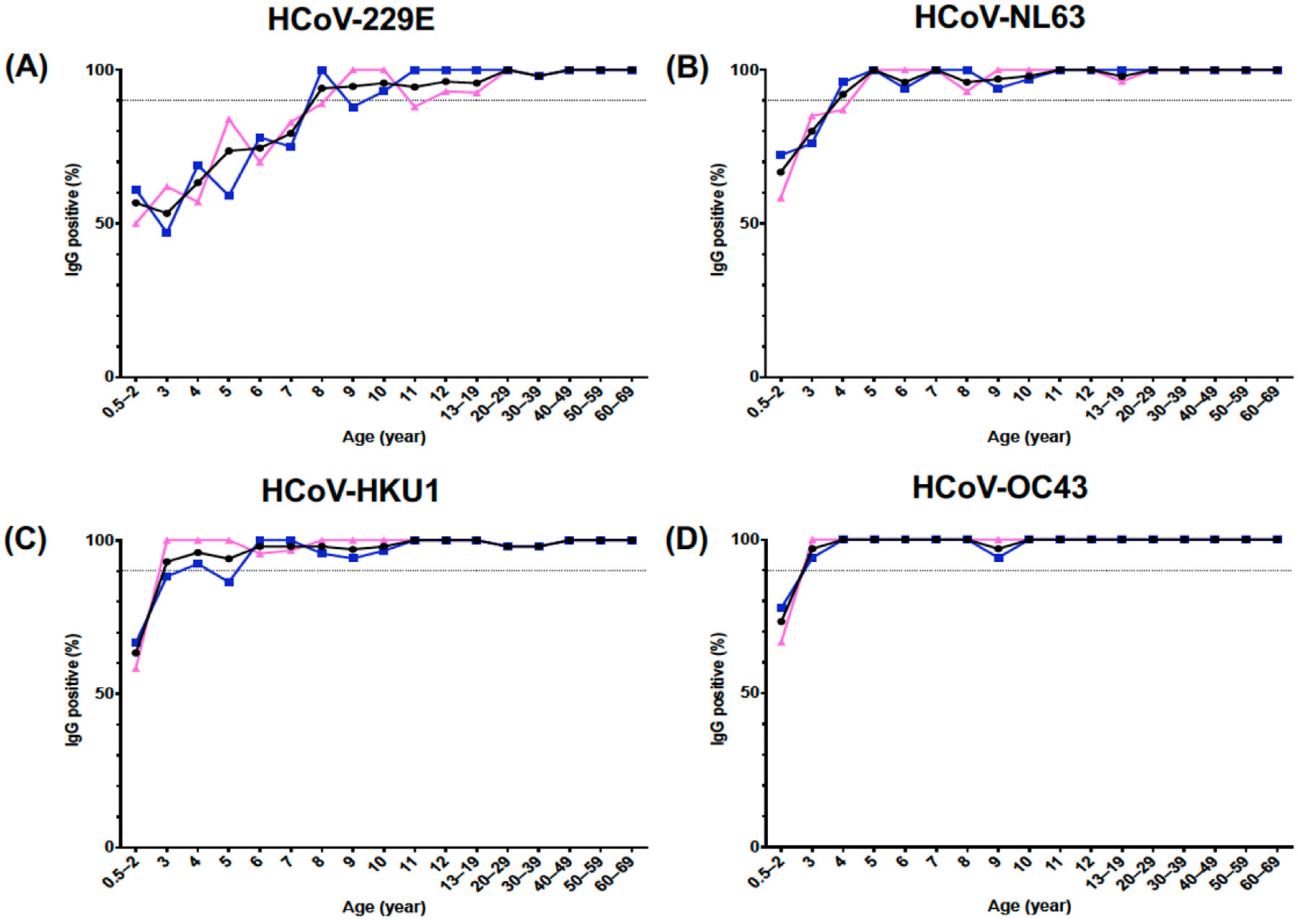
Seroprevalence of the four endemic HCoVs among different age groups in Japan.

The distribution of IgG antibody positivity among age groups is shown in Figure 1. The IgG seropositivity prevalence against four endemic HCoV spike ectodomain proteins was 50-70% in children younger than 2 years. The IgG seropositivity for HCoV-NL63, HCoV-HKU1, and HCoV-OC43 reached 90% at the age of 3-4 years, whereas that for HCoV-229E showed 50-70% at the same age. A total of 90% IgG seropositivity for HCoV-229E was achieved at 8 years of age. The seropositivity of the four endemic HCoVs remained high until 60 years of age.

IgG antibody positivity against (A) HCoV-229E, (B) HCoV-NL63, (C) HCoV-HKU1, and (D) HCoV-OC43 spike ectodomain. Black indicates the total (male and female) study individuals. Blue and red indicate male and female samples, respectively. The line shows 90% IgG antibody positivity. HCoV, human coronaviruses; Ig, immunoglobulin.

### Analysis of SARS-CoV-2 seropositive samples

**Figure 2:**
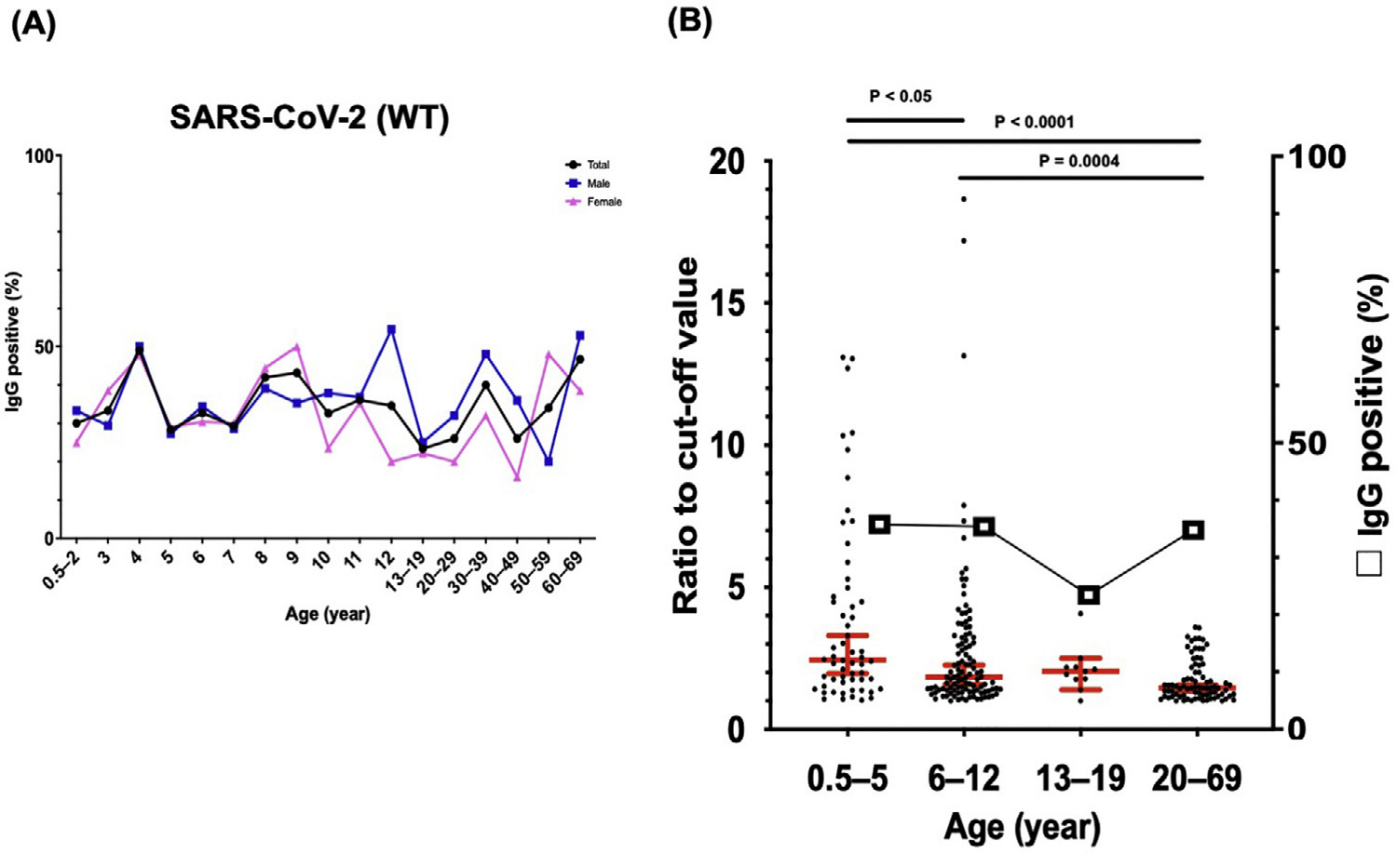
Seropositivity for SARS-CoV-2 WT using samples collected before the COVID-19 pandemic. (a) IgG antibodies positivity against the SARS-CoV-2 WT spike ectodomain. (b) Comparing antibody levels (ratio to cut-off value) using reactive samples against SARS-CoV-2 WT among different age groups (age 0.5-5, 6-12, 13-19, and 20-69 years). The horizontal lines show the mean value and 95% confidence interval. The Mann–Whitney U test was used to compare the antibody levels between each group and *P*-values. Ig, immunoglobulin; WT, wild-type.

SARS-CoV-2 WT seropositivity was shown in 258 (34.5%) samples and in a transition of approximately 20-50% among all age groups (Table 1 and Figure 2A). We also analyzed antibody levels in each age group using SARS-CoV-2 WT–reactive samples. IgG seropositivity was approximately 35% among the three age groups (0.5-5, 6-12, and 20-69 years) and 23% among individuals aged 13-19 years (Figure 2B). The antibody levels against SARS-CoV-2 WT in individuals aged 0.5-5 years were significantly higher than those in the other two groups (age 6-12 years and 20-69 years). In addition, study individuals aged 6-12 years showed significantly higher antibody levels than those aged 20-69 years.

**Figure 3:**
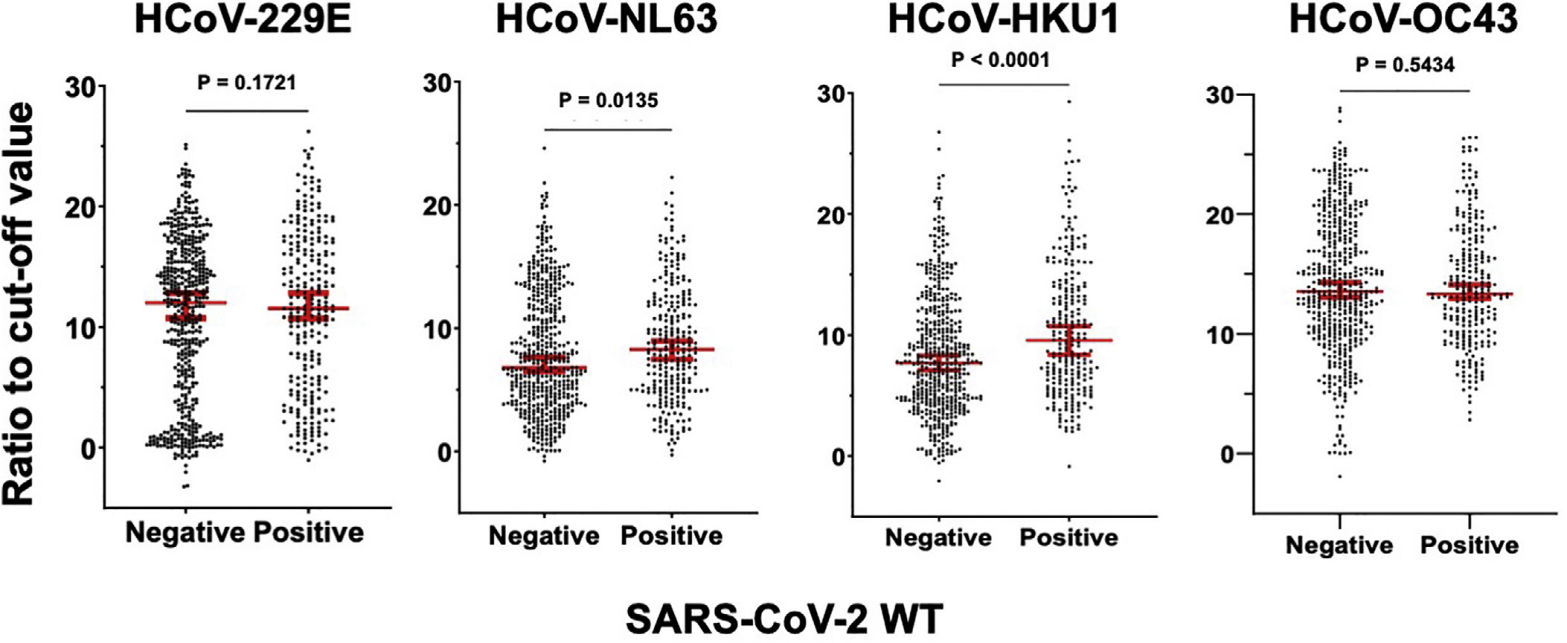
Comparison of antibody levels in four endemic HCoVs in SARS-CoV-2–reactive and –non-reactive samples.

Moreover, we investigated antibody levels (ratio to cut-off value) against four endemic HCoVs in a subset of samples from SARS-CoV-2–reactive (n = 255) and –non-reactive samples (n = 492) (Figure 3). The antibody levels against HCoV-NL63 and HCoV-HKU1 were significantly higher in individuals with SARS-CoV-2–reactive antibodies than in individuals who did not have SARS-CoV-2–reactive antibodies.

Seroreactivity to HCoV-229E, HCoV-NL63, HCoV-HKU1, and HCoV-OC43 was analyzed by performing enzyme-linked immunosorbent assay against the spike ectodomain proteins of each virus using SARS-CoV-2 positive (n = 258) and negative (n = 489) samples. The y-axis shows the ratio to the cut-off value of each antigen. The horizontal lines show the mean value and 95% confidence interval. The results were compared using the Mann–Whitney U test and *P*-values. HCoV, human coronaviruses; WT, wild-type.

### Neutralization assay using moderate to highly reactive samples against SARS-CoV-2 wild-type

The 62 moderate to highly reactive samples (ratio to cut-off-value ≥3) against SARS-CoV-2 WT showed the following distribution across different age groups based on the ELISA results and the investigated neutralization capability of plaque reduction neutralization test: 0.5-5 years (n = 23), 6-12 years (n = 31), 13-19 years (n = 1), and 20-69 years (n = 7). None of the 62 reactive samples exhibited a 50% neutralization inhibition rate.

## Discussion

This study demonstrated the seroprevalence of the four endemic HCoVs in Japan and the antibody reactivity of children and adults against SARS-CoV-2 using serum samples collected before the COVID-19 pandemic. The seroprevalence analysis of the four endemic HCoVs revealed that 50-70% of children aged <2 years harbored IgG antibodies against four endemic HCoV spike ectodomains. Subsequently, 90% samples obtained from children aged 3-4 years were seropositive for HCoV-NL63, HCoV-HKU1, and HCoV-OC43. In contrast, 90% seropositivity for HCoV-229E was observed in children aged 8 years. In the SARS-CoV-2 reactive analysis, 20-50% samples from children and adults were IgG seropositive against SARS-CoV-2 WT, and the antibody levels against SARS-CoV-2 WT in children were significantly higher than those in adults. However, sample reactivity against SARS-CoV-2 did not confirm neutralization capability.

Most children are infected with the four endemic HCoVs [[11], [12], [13], [14], [15]]. The seropositivity against the four endemic HCoVs plateaus by the age of 10 years, although the seroprevalence of each HCoV differs by region [[11], [12], [13],^15^,^24^]. In this study, 90% of samples from children aged 3-4 years tested seropositive for three HCoVs (HCoV-NL63, HCoV-HKU1, and HCoV-OC43), and for one remaining HCoV-229E, seropositivity was achieved by 8 years of age. This finding is supported by that of a previous study in Yamagata, a neighboring area of the sampling site in this study; the study showed that HCoV-229E caused the fewest infections among other endemic HCoVs [25]. Based on these findings, we propose that seropositivity for HCoV-229E showed a slower increase in our study area owing to fewer opportunities for infection with HCoV-229E than with other endemic HCoVs. Furthermore, the findings of this study are consistent with those of previous studies, in which the serological analyses of samples from children in the Netherlands, Canada, and the Philippines indicated that HCoV-229E had a slower increase in antibody positivity rates than the other three HCoVs [11,15,24]. Our previous study in the Philippines showed that HCoV-NL63, HCoV-HKU1, and HCoV-OC43 but not HCoV-229E were detected in children hospitalized with pneumonia [26]. On the other hand, another study in China indicated that antibody positivity for HCoV-229E showed the highest positive rate among the four endemic HCoVs in the age group 1-3 years [12]. Therefore, the increase in the positivity rate of antibodies to each of the four endemic HCoVs may differ based on the circulation patterns of HCoVs in each region.

The clinical symptoms of SARS-CoV-2 infection in most children are mild or asymptomatic [16]. To elucidate the underlying reason for this, we analyzed the seroreactivity against SARS-CoV-2 WT using child and adult serum samples collected before the COVID-19 pandemic. We observed 20-50% seropositivity in all age groups and antibody levels in children; antibody levels in the 0.5-5-year-old group were significantly higher than those in the adult group. Higher anti-SARS-CoV-2 antibody levels had been reported in children than in older individuals in Finland [22]. These results suggest that children may develop cross-reactive antibodies against coronaviruses owing to earlier infections with other endemic HCoVs.

This study showed that the antibody levels against HCoV-NL63 and HCoV-HKU1 were significantly higher in SARS-CoV-2 reactive samples than in SARS-CoV-2–non-reactive samples. Angiotensin-converting enzyme 2 and TMPRSS2 have been identified as receptors of HCoV-NL63 and HCoV-HKU1, respectively [27,28]. It is known that SARS-CoV-2 is also a receptor for these factors [29]. Thus, there may be a relationship between the receptors and antibody levels for two endemic HCoVs (HCoV-NL63 and HCoV-HKU1) and SARS-CoV-2. However, SARS-CoV-2 cross-reactive samples in this study did not show a neutralization capability against the SARS-CoV-2 live virus. Most samples reactive against SARS-CoV-2 that were collected before the COVID-19 pandemic hardly had any neutralizing antibodies [11,19,20]. Therefore, factors other than antibodies may also be involved. Indeed, children mount higher innate antiviral responses [30] and show a lower expression of angiotensin-converting enzyme 2 than adults [31]. Thus, several factors may be attributed to mild or asymptomatic COVID-19 infection in children, including immunocompetence and receptor expression.

There are several limitations to this study. First, we could not perform a deep investigation of the 13-19-year-old group and serological analyses using samples from those above 70 years of age. COVID-19 leads to more severe symptoms in the elderly population. Therefore, further studies should perform detailed analyses in these age groups. In addition, we could not identify epitopes of reactive samples against the SARS-CoV-2 spike ectodomain. Reactive samples mainly harbor antibodies against the SARS-CoV-2 S2 domain, which has a high homology between HCoVs and SARS-CoV-2, whereas the neutralization capability is mainly associated with the receptor-binding region of the S1 domain. This may explain why samples reactive against SARS-CoV-2 in this study could not neutralize SARS-CoV-2.

In conclusion, this study showed differences in seroprevalence among four endemic HCoVs. In particular, the seroprevalence of HCoV-229E was lower than that of the other three HCoVs. In addition, approximately 35% of the samples showed IgG seropositivity against SARS-CoV-2. The antibody levels against the SARS-CoV-2 WT varied among different age groups, especially children with high antibody levels. However, reactive samples against the SARS-CoV-2 WT spike ectodomain protein that were collected before the COVID-19 pandemic did not show neutralization capability against the SARS-CoV-2 live virus. The findings of this study may deepen our understanding of the clinical symptoms of COVID-19 and coronavirus diseases in children and adults.

## Declarations of competing interest

The authors have no competing interests to declare.
